# Impact of opioid use on patients undergoing screening colonoscopy according to the quality of bowel preparation

**DOI:** 10.1002/jgh3.12288

**Published:** 2019-12-09

**Authors:** Lois Lamerato, Eric Wittbrodt, Manpreet Kaur, Catherine Datto, Sumit Singla

**Affiliations:** ^1^ Department of Public Health Sciences Henry Ford Health System Detroit Michigan USA; ^2^ AstraZeneca Wilmington Delaware USA; ^3^ Department of Gastroenterology Henry Ford Health System Detroit Michigan USA

**Keywords:** analgesics, opioid, bowel preparation solutions, colonoscopy

## Abstract

**Aims:**

Constipation associated with opioid therapy for chronic pain may negatively impact colonoscopy success. This retrospective, observational study using administrative data and electronic medical records evaluated the impact of opioid use on colonoscopy outcomes.

**Methods and Results:**

Procedural codes were used to identify patients who had a screening colonoscopy at two Henry Ford Health System centers (January 2015–December 2016). All patients had completed a standard uniform bowel preparation protocol. Medication orders and filled prescriptions were used to identify patients with a history of opioid use during the 28 days preprocedure (exposed) and a matched random sample of presumptive opioid nonusers (unexposed). Electronic medical records were reviewed for colonoscopy procedure data and outcomes.

The exposed and unexposed groups included 964 and 1054 patients, respectively. Inadequate bowel preparation was significantly more common in the exposed *versus* unexposed group (18.5% *vs* 12.7%; *P* < 0.001). In the exposed and unexposed groups, 97.1 and 98.0% of colonoscopy procedures were completed, respectively (*P* = nonsignificant). Total procedure time was slightly increased for the exposed *versus* unexposed group (23.8 *vs* 22.5 min; *P* = 0.039). Polyp identification and cancer diagnosis were similar between groups. Prolonged sedation occurred in three patients in the exposed group and none in the unexposed group. Procedural complications were rare, but the incidence was significantly greater in the exposed *versus* unexposed group (1.3% *vs* 0.2%; *P* < 0.01).

**Conclusions:**

Opioid exposure was associated with significant reductions in the quality of preprocedure bowel preparation and an increased risk of complications in patients undergoing colonoscopy.

## Graphical abstract

Constipation associated with opioid therapy for chronic pain may negatively impact colonoscopy success. This retrospective, observational, cohort study using administrative data and electronic medical records showed that opioid exposure was associated with significant reductions in the quality of preprocedure bowel preparation and an increased risk of complications in patients undergoing colonoscopy.

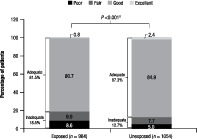

## Introduction

Colonoscopy is recommended in current guidance from the US Multi‐Society Task Force on Colorectal Cancer as a first‐tier screening test for colorectal cancer (CRC)^1^ and is the most commonly used test to screen for CRC.^2^ In 2012, approximately 6.5 million screening colonoscopies were performed in the United States.^2^ The use of screening colonoscopies is associated with an estimated 87% median reduction in the lifetime risk of CRC‐related death.^3^ Based on improvements in CRC outcomes with screening colonoscopies, the National Colorectal Cancer Roundtable initiative has proposed a goal of increasing the CRC screening rate among adults 50 years of age and older to 80% in the United States.^4^

A reduction in the risk of advanced and fatal CRC using screening colonoscopies depends on the detection of adenomas,^5^ and the diagnostic accuracy and safety of colonoscopy for detecting colonic lesions depend, in part, on the quality of bowel preparation.^6^, ^7^ Bowel preparation has been shown to be suboptimal in approximately 10–35% of colonoscopies,^8^, ^9^ and suboptimal bowel preparation may, in part, be due to the presence of chronic constipation and use of medications that slow gut motility.^10^ Inadequate bowel preparation has been associated with a 44% decrease in the chance of detecting early colonic polyps and a 23% decrease in the chance of detecting advanced polyps.^6^ Along with contributing to missed adenomas, inadequate bowel preparation may be associated with an increased risk of procedure‐related complications,^11^ as well as increased costs, increased procedure times, canceled procedures, and the need for repeat examination.^12^

Chronic pain is one of the most common reasons for seeking medical care in the United States, and it is estimated that up to 20% of patients with noncancer chronic pain are prescribed opioid analgesics in the outpatient setting.^13^, ^14^, ^15^ Constipation has been reported in approximately 40–80% of patients taking opioids for chronic pain.^16^, ^17^ Several large studies have shown that prior exposure to opioids (at both low and high doses) represents an independent risk factor for poor bowel preparation.^8^, ^18^

The current study was conducted to evaluate not only the quality of bowel preparation for patients on opioid therapy but also the impact of opioid use on colonoscopy outcomes.

## Methods

### *Study design*

This retrospective, observational study used administrative and electronic medical record (EMR) data from patients undergoing colonoscopy at two medical centers in the Henry Ford Health System (HFHS). Colonoscopy procedures, performed by practicing physicians or fellows in training, were limited to these two sites to minimize variability in provider behavior and provider documentation practices. The majority (90%) of patients undergoing colonoscopy in the HFHS receive a standard split bowel preparation (polyethylene glycol 3350 and electrolytes oral solution [Braintree Laboratories, Inc. Braintree, MA, USA] or sodium sulfate, potassium sulfate, and magnesium sulfate oral solution [Braintree Laboratories, Inc.]). Patients who had a previous poor bowel preparation or are believed by the clinician to be at high risk for a poor bowel preparation receive an extended, two‐day preparation. No alterations in the bowel preparation procedure are made for patients taking opioids. Colonoscopies were performed using standard procedures at the two medical centers, using either air or carbon dioxide for insufflation. This study was approved by the HFHS Institutional Review Board on November 6, 2016.

### *Patient selection*

Screening colonoscopies in adults (≥18 years of age) performed in 2015 and 2016 were identified using Current Procedural Terminology, 4th edition (CPT‐4; American Medical Association) codes. Patients with diagnostic and treatment‐related colonoscopy codes were not included in order to minimize clinical factors that might influence the colonoscopy procedure. The codes included were: 45380 (colonoscopy, flexible, with biopsy single or multiple), 45385 (colonoscopy, flexible proximal to splenic flexure, with removal of tumor), G0105 (CRC screening colonoscopy on individual at high risk), and G01021 (colorectal screen colonoscopy on individual not meeting high‐risk criteria). In the event that a patient had two colonoscopies during the study time frame, the first procedure was used as the study colonoscopy.

### *Assessment of opioid exposure*

Medication orders and prescriptions filled during a period of six months prior to the colonoscopy date were reviewed for assessment of opioid exposure. Patients who had documented opioid use within the 28 days prior to the colonoscopy date were classified as presumptive opioid users (exposed group). In identifying the presumptive exposed group, patients with a medication order or filled prescription for an opioid 42 days prior to the date of colonoscopy were included for subsequent EMR review to confirm the opioid exposure window based on the assumption that an order or prescription for an opioid for chronic pain would be for at least a two‐week supply of medication. Patients with an opioid order only on the date of the colonoscopy were not considered part of the exposed group due to the use of opioids for sedation during the procedure. In addition, patients with historical exposure to opioids (i.e. with evidence of exposure >42–180 days prior to the procedure) but no recent (within 42 days of the procedure) evidence of opioid exposure were excluded from the study. The remaining patients were presumed unexposed to opioid medication. Additional exclusion criteria were a documented history of CRC or exposure to the mixed opioid agonist–antagonists buprenorphine, butorphanol, or nalbuphine within 180 days preprocedure.

**Figure 1:**
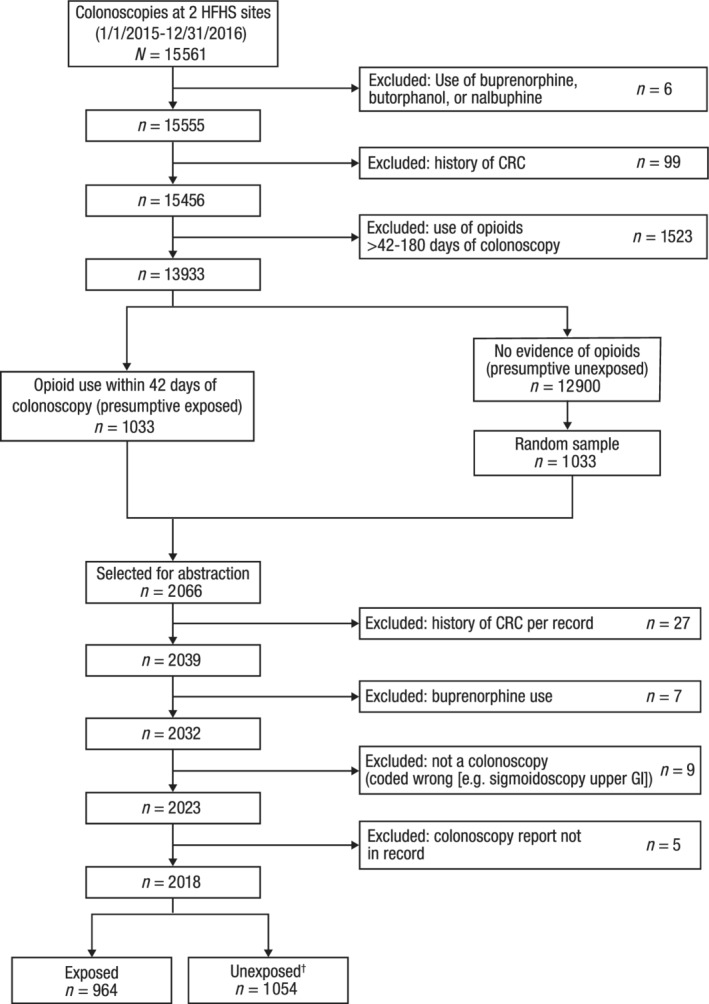
Final population. CRC, colorectal cancer; GI, gastrointestional. ^†^21 patients were reclassified as opioid unexposed based on chart review.

A total of 15 561 colonoscopy records were retrieved from the EMRs. After excluding patients based on prespecified criteria, as shown in Figure 1, a final analysis population was determined, which included 964 patients exposed to opioids and 1054 patients unexposed to opioids.

### *Data abstraction*

Study data were collected and managed using Research Electronic Data Capture (REDCap) electronic data capture tools hosted at HFHS.^19^ REDCap is a secure, web‐based application designed to support data capture for research studies, providing: (i) an intuitive interface for validated data entry, (ii) audit trails for tracking data manipulation and export procedures, (iii) automated export procedures for seamless data downloads to common statistical packages, and (iv) procedures for importing data from external sources. The electronic medical record, Epic (Copyright 2018 Epic Systems Corporation) was reviewed to abstract opioid exposure, opioid use (specific drug, indication, formulation, dosage, duration of exposure), and data on colonoscopy procedures and outcome variables.

### *Quality checks*

Two quality assurance studies were conducted to assess the accuracy of data abstraction, in addition to two pilot studies, prior to finalizing the study variables and database containing the study population. The quality assurance studies involved selection of a random sample of 5% of abstracted cases, which were subjected to review by a second abstractor, and the results were compared.

### *Outcome measures*

The primary objective of this study was to compare the incidence of failed or inadequate colonoscopy in patients exposed to opioids with those unexposed to opioids. Secondary objectives included comparing the quality of bowel preparation (recorded in the procedure report at the time of the colonoscopy by the physician), intraprocedural time, incidence of repeat (rescheduled) colonoscopy, complications of colonoscopy, and the proportion of patients with abnormal findings (polyps, etc.) in the presumed exposed and unexposed groups.

Bowel preparation data were collected during the colonoscopy procedure and recorded by the endoscopist as excellent, good, fair, or poor and subsequently dichotomized into adequate (excellent and good) *versus* inadequate (fair and poor) for analysis. Generally, the criteria for adequacy of bowel preparation included the ability to visualize the colonic mucosa and to achieve cecal intubation. Other information retrieved from the EMRs included the time to cecum, total procedural time, complications, recommendation and date of any repeat colonoscopy, and findings (for study and repeat colonoscopies: lesions, tissue sampling, pathology reports). Procedure‐related complications were defined as lack of completion, prolonged sedation, bleeding, perforation, or other complications. Colonoscopy completion was assessed as a separate outcome. Incidents that were considered unrelated to the colonoscopy were not included in this assessment. Unplanned hospitalization following colonoscopy was also captured.

### *Statistical analyses*

Demographic characteristics of the opioid‐exposed and ‐unexposed groups were compared using chi‐squared test or Fisher's exact test. As noted previously, bowel preparation was dichotomized into adequate (excellent or good) and inadequate (fair or poor) categories. These were introduced into a binary logistic regression model that included gender, ethnicity, age category, and opioid exposure. Unadjusted and adjusted odds ratios (ORs) were then calculated, together with *P* values.

## Results

### *Patients*

When comparing the opioid‐exposed and ‐unexposed groups, mean age was comparable, and there were no differences in gender distribution (Table 1). However, significant differences were observed between age categories for the exposed and unexposed groups, with a higher proportion of patients in the exposed group in the youngest subset (18–49 years of age). In addition, the proportion of Asians was higher in the unexposed group than in the exposed group.

**Table 1:** Baseline characteristics for patients exposed to opioids and patients unexposed to opioids†

Characteristic	*n*	Exposed	Unexposed	*P* value
Gender				
Male	884	414 (46.8)	470 (53.2)	0.457
Female	1134	550 (48.5)	584 (51.5)	
Age, years, mean		58.0	59.1	0.065
Age category, years				<0.001
18–49	307	179 (58.3)	128 (41.7)	
50–59	705	311 (44.1)	394 (55.9)	
60–69	693	309 (47.0)	384 (53.0)	
≥70	349	165 (47.3)	184 (52.7)	
Race				<0.001
White	741	352 (47.5)	389 (52.5)	
African American	995	506 (50.9)	489 (49.1)	
Hispanic	62	29 (46.8)	33 (53.2)	
Asian	35	8 (22.9)	27 (77.1)	
Other/unknown	185	69 (37.3)	116 (62.7)	

† Values are *n* (%) unless otherwise specified.

Approximately one‐quarter of the exposed patients used opioids for less than one week, whereas 61% used opioids for at least 28 days. A total of 39% of exposed patients had used opioids for more than four months. When opioid exposure was evaluated by demographic characteristics, a higher proportion of patients in the youngest age category (18–49 years of age) used opioids for less than one week to 27 days compared with the other age categories (*P* < 0.001). In addition, a higher proportion of women used opioids for at least 28 days (*P* < 0.05). The most commonly used opioids included hydrocodone/hydrocodone + acetaminophen (46.8% of exposed patients), morphine/morphine sulfate (25.7%), tramadol (20.2%), and fentanyl (19.5%). During the four‐week period prior to colonoscopy, 58% of patients exposed to opioids received one opioid, 28% received two opioids, and 14% received three or more opioids. The most common indications for opioid use were abdominal pain (25%), back pain (22%), and arthritis or joint pain (20%).

### *Colonoscopy characteristics and outcomes*

**Figure 2:**
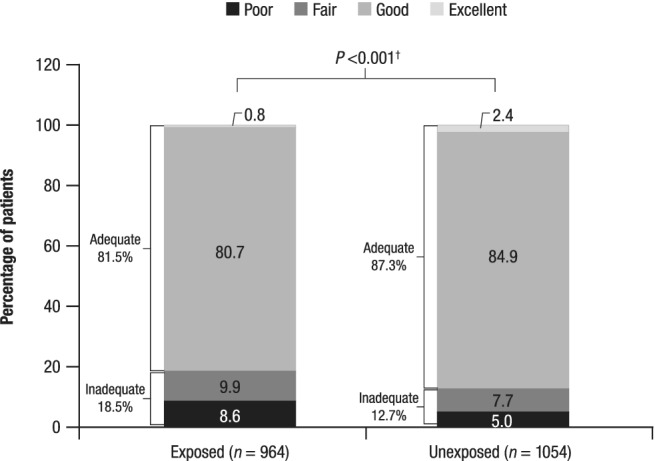
Adequacy of bowel preparation for patients exposed to opioids and patients unexposed to opioids. ^†^Exposed *versus* unexposed for bowel preparation quality (excellent, good, fair, poor) and adequacy (adequate, inadequate).

There was a significant difference between the opioid‐exposed and ‐unexposed groups for the quality of bowel preparation, with lower proportions of opioid‐exposed patients than unexposed patients having excellent (0.8% *vs* 2.4%) or good (80.7% *vs* 84.9%) preparations (Fig. 2). Bowel preparation was considered adequate (excellent or good) in 81.5% of patients exposed to opioids compared with 87.3% of patients unexposed to opioids (*P* < 0.001; Fig. 2). The rate of completed colonoscopies was high (>97%) in both groups, and time to cecum was similar. The overall procedure time (excluding patients for whom the procedure was unable to be completed) was slightly increased for patients in the opioid‐exposed group compared with the unexposed group (mean, 23.9 *vs* 22.6 min; *P* = 0.039; Table 2); however, it should be noted that the time was not documented in 18 and 17% of EMRs, respectively.

**Table 2:** Summary of colonoscopy procedures and outcomes for patients exposed to opioids and patients unexposed to opioids†

	Exposed (*n* = 964)	Unexposed (*n* = 1054)	*P* value
Colonoscopy completed	936 (97.1)	1033 (98.0)	0.184
Time to cecum, min, mean	7.9	7.5	0.149
Missing data	98 (10.1)	108 (10.2)	
Colonoscopy procedure time, min, mean	23.9	22.6	
Missing data	175 (18.1)	178 (16.9)	0.039
Polyps identified, patients	518 (53.7)	580 (55.0)	0.589
Number of polyps removed, mean	2.3	2.5	0.162
Cancer diagnosis‡	11 (1.1)	6 (0.6)	0.160
Repeat colonoscopy recommended	63 (6.5)	67 (6.4)	0.866
Reason for repeat colonoscopy			
Inadequate bowel preparation	40 (53.3)	35 (46.7)	0.194
Other	23 (41.8)	32 (58.2)	
Repeat colonoscopy completed within 6 months	42 (66.7)	47 (70.1)	0.669
Prolonged sedation	3 (0.3)	0 (0)	0.109
All complications (patients)	15 (1.6)	3 (0.3)	<0.01
Procedure‐related complications	13 (1.3)	2 (0.2)	<0.01
Type of procedure‐related complication§			
Abdominal pain	8 (0.8)	2 (0.2)	0.055
Bleeding	4 (0.4)	0 (0)	0.052
Perforation	1 (0.1)	0 (0)	0.487
Other	5 (0.5)	2 (0.2)	0.269
Unplanned hospitalization following colonoscopy	4 (0.4)	1 (0.1)	0.199

† Values presented are *n* (%), unless otherwise specified.

‡ Colorectal cancers with the exception of one case of metastatic prostate cancer.

§ Patients may have experienced more than one type of procedure‐related complication.

There were no differences between groups for the rate of polyp detection or polyp removal (Table 2). Of the 1908 patients who had a polyp identified, 1078 had tissue removed, which was determined as adenomatous in 726 cases. A diagnosis of CRC was made in 16 patients and metastatic prostate cancer in one patient. The need for repeat colonoscopy was similar in the opioid‐exposed and ‐unexposed groups (6.5% *vs* 6.3%). Inadequate bowel preparation was the reason for repeat colonoscopy in 75 patients (exposed group, *n* = 40; unexposed group, *n* = 35; *P* = 0.194).

The rate of procedural complications was low in both groups but was significantly more frequent in patients with prior opioid exposure compared with patients unexposed to opioids (1.3% *vs* 0.2%; *P* < 0.01; Table 2). Complications in three patients were unrelated to the procedure; these included an incident of ulcerative colitis (previously diagnosed) requiring intravenous steroids (exposed group), bradycardia during the procedure (unexposed group), and an anesthesia‐related complication (exposed group). Rates of unplanned hospitalization following colonoscopy were low in both groups.

Irrespective of whether patients had adequate or inadequate bowel preparation, there were no differences in the requirement for repeat colonoscopy, prolonged sedation, and unplanned hospitalizations following colonoscopy between the opioid‐exposed and opioid‐unexposed groups (Table 3). In the subset of patients who had an adequate bowel preparation, patients who were exposed to opioids had a higher frequency of procedure‐related complications and bleeding than those who were unexposed to opioids. The few patients who reported complications of bleeding or perforation had no complications noted during the procedure. All patients with bleeding or perforation complications had polyps removed and subsequently presented to the emergency room with postprocedure symptoms of hematochezia or hematemesis. No invasive interventions or transfusions were required. In the subset of patients with inadequate bowel preparation, there were no significant differences in complications between patients exposed to opioids and those unexposed to opioids. In this subset, inadequate bowel preparation necessitated repeat colonoscopy in 89.7% of patients in the opioid‐exposed group and 86.5% of patients in the unexposed group.

**Table 3:** Summary of colonoscopy procedures and outcomes for patients exposed to opioids and patients unexposed to opioids†

Procedure and outcomes	Exposed	Unexposed	*P* value
*Adequate bowel preparation*	*n = 786*	*n = 920*	
Repeat colonoscopy recommended	24 (3.1)	30 (3.3)	0.811
Reason for repeat colonoscopy			0.443
Inadequate bowel preparation	5 (20.8)	2 (10.0)	
Other	19 (79.2)	27 (90.0)	
Prolonged sedation	2 (0.3)	0 (0)	0.212
Procedure‐related complications	8 (1.0)	1 (0.1)	<0.01
Type of procedure‐related complication‡			
Abdominal pain	4 (0.5)	1 (0.1)	0.187
Bleeding	4 (0.5)	0 (0)	<0.05
Perforation	0 (0)	0 (0)	NA
Other	3 (0.4)	1 (0.1)	0.340
Unplanned hospitalization following colonoscopy	2 (0.3)	1 (0.1)	0.560
*Inadequate bowel preparation*	*n* = 178	*n* = 134	
Repeat colonoscopy recommended	39 (21.9)	37 (27.6)	0.245
Reason for repeat colonoscopy			0.733
Inadequate bowel preparation	35 (89.7)	32 (86.5)	
Other	4 (10.3)	5 (13.5)	
Prolonged sedation	1 (0.6)	0 (0)	0.999
Procedure‐related complications	5 (2.8)	1 (0.7)	0.242
Type of procedure‐related complication‡			
Abdominal pain	4 (2.2)	1 (0.7)	0.396
Bleeding	0 (0)	0 (0)	NA
Perforation	1 (0.6)	0 (0)	0.999
Other	2 (1.1)	1 (0.7)	0.999
Unplanned hospitalization following colonoscopy	2 (1.1)	0 (0)	0.508

NA, not applicable.

† Values presented are *n* (%).

‡ Patients may have experienced more than one type of procedure‐related complication.

From the binary logistic regression model, it was found that exposure to opioids and male gender were both independently associated with a significantly reduced likelihood of adequate bowel preparation (Table 4). No influence of ethnicity or age category was noted.

**Table 4:** Logistic regression for adequacy of bowel preparation

	Unadjusted OR (95% CI)	*P* value	Adjusted OR (95% CI)	*P* value
Opioid exposed *versus* unexposed	0.64 (0.50–0.82)	<0.01	0.64 (0.50–0.81)	<0.001
Male *versus* female	0.70 (0.55–0.89)	<0.01	0.69 (0.54–0.88)	<0.01
Ethnicity				
White (reference)	1.00			
African American	0.88 (0.67–1.14)	0.33		
Hispanic	0.79 (0.40–1.57)	0.50		
Asian	2.81 (0.67–11.88)	0.16		
Other/unknown	0.92 (0.59–1.43)	0.71		
Age category†	1.05 (0.92–1.19)	0.47		

CI, confidence interval; OR, odds ratio.

† *Age categories*: 18–49 years, 50–59 years, 60–69 years, and ≥ 70 years.

## Discussion

The aim of the current study was to compare colonoscopy outcomes between patients who were opioid users within a relatively close time frame to the colonoscopy procedure and those who had not been exposed to opioids; thus, patients with evidence of current opioid exposure within the month prior to the procedure were selected for inclusion in the analysis, while those with only historical exposure were excluded. These selection criteria for opioid exposure were considered sufficient to have an impact on gastrointestinal motility, while the criteria for unexposed patients likely provided sufficient time since the termination of opioid use to allow for complete washout of the opioid effect on motility and any corresponding effect on bowel preparation. Based on these criteria, 7.4% of patients who had undergone screening colonoscopy were included in the opioid‐exposed group. This rate was comparable to that in a previous retrospective study of outpatients undergoing colonoscopy, in which 8.6% were identified as regular opioid users.^18^

Although there was no significant difference in the proportion of completed colonoscopies for patients in the opioid‐exposed and opioid‐unexposed groups, opioid exposure was associated with a significantly lower proportion of patients with adequate bowel preparation compared with the opioid‐unexposed group. Male gender was also independently associated with poorer bowel preparation quality prior to screening colonoscopy. Findings from previous studies support the association of opioid exposure and male gender with poor bowel preparation quality.^8^, ^18^, ^20^ In addition, the proportion of patients who were unexposed to opioids with adequate bowel preparation was comparable in this study (87.3%) with that reported in previous studies (75.6% and 90.7%).^8^, ^18^ In the current study, the use of opioids was also shown to have an impact on other colonoscopy outcomes, beyond the adequacy of bowel preparation. The time to cecum was comparable regardless of opioid exposure; however, those patients exposed to opioids had a slightly longer overall procedure time, but this difference was not clinically significant. It has been suggested that patients with a history of opioid use are more difficult to sedate,^21^ which could potentially contribute to this observed difference in procedure time. Furthermore, although the rate of procedure‐related complications was low in both groups, procedural complications were observed in a significantly higher proportion of patients exposed to opioids compared with unexposed patients.

In this study, only a small proportion of patients (approximately 6.5%) required repeat colonoscopy, which was necessitated by inadequate bowel preparation in approximately 50% of cases for both patients exposed and unexposed to opioids. When comparing adequate and inadequate bowel preparation, there were no differences in the need for repeat procedures according to opioid exposure.

CRC is the third leading cause of cancer‐related death in the United States^22^; however, early diagnosis allows for identification of early‐stage, localized disease, which has a 5‐year survival rate of 90%.^23^ Colonoscopy is the current standard for screening for CRC,^1^ and more than 6 million screening colonoscopies are performed in the United States in a single year.^2^ Adequate bowel preparation is essential for colonoscopy success.^6^, ^7^, ^11^ The presence of chronic constipation may have a negative impact on bowel preparation,^10^ and the use of opioids for chronic noncancer pain is often associated with constipation.^16^, ^17^ To achieve the current goal of increasing screening colonoscopies,^4^ colonoscopy suite efficiency will need to be maximized, while the wasted resources (e.g. endoscopy suite time; nurse, anesthesiologist, and endoscopist time; medical supplies/equipment for colonoscopy) associated with incomplete and repeated procedures will need to be minimized. In the HFHS, repeat colonoscopy is also associated with substantial additional costs, with total uninsured costs listed as $1730 for a screening colonoscopy, $1850 for colonoscopy with biopsy, and $1950 for colonoscopy with polyp removals.^24^ Maximizing colonoscopy suite efficiency will require identifying patients with known risk factors for inadequate bowel preparation, including opioid use, who may benefit from more aggressive bowel preparation^12^ or from patient education regarding the methods and benefits of adequate bowel preparation.^25^

The strengths of this study included the relatively large sample size and high likelihood of consistent procedural approach because data were restricted to two centers within the same health system. There were also some potential limitations. It is well established that patients with diabetes experience issues with bowel preparation^20^, ^26^ and may require specific preparation regimens.^27^ Use of other medications and patients’ functional status may also affect bowel motility; however, comorbidities (e.g. diabetes), other medications, and patients’ functional status were not explored as potential modifying factors in the current analysis. In addition, although screening colonoscopies were selected during data capture based on CPT‐4 codes, potential coding irregularities may have affected the data included. In addition, there were fewer Asian patients and more patients in the youngest age category in the opioid‐exposed group; however, in the regression analysis, neither ethnicity nor age category was found to be associated with adequate bowel preparation. This is in agreement with previous studies evaluating bowel preparation.^28^

This study supports the results of previous studies^8^, ^18^ showing the negative impact of opioid use on bowel preparation but also indicates that opioid use may be associated with more procedure‐related complications and slightly longer procedure times. These differences were generally relatively small. Nevertheless, these results suggest that additional measures to optimize bowel preparation prior to colonoscopy may be necessary for some patients taking opioids. Additional research is needed to determine which methods of bowel preparation or other measures (e.g. educational interventions) may be most beneficial for chronic opioid users.
